# Spontaneous Brain Activity Did Not Show the Effect of Violent Video Games on Aggression: A Resting-State fMRI Study

**DOI:** 10.3389/fpsyg.2017.02219

**Published:** 2018-01-12

**Authors:** Wei Pan, Xuemei Gao, Shuo Shi, Fuqu Liu, Chao Li

**Affiliations:** ^1^Faculty of Psychology, Southwest University, Chongqing, China; ^2^Institute of Psychology, Chinese Academy of Sciences, Beijing, China; ^3^Department of Psychology, University of Chinese Academy of Sciences, Beijing, China

**Keywords:** violent video game, aggression, resting state fMRI, amplitude of low-frequency fluctuations (ALFF), fractional ALFF

## Abstract

A great many of empirical researches have proved that longtime exposure to violent video game can lead to a series of negative effects. Although research has focused on the neural basis of the correlation between violent video game and aggression, little is known whether the spontaneous brain activity is associated with violent video game exposure. To address this question, we measured the spontaneous brain activity using resting-state functional magnetic resonance imaging (fMRI). We used the amplitude of low-frequency fluctuations (ALFF) and fractional ALFF (fALFF) to quantify spontaneous brain activity. The results showed there is no significant difference in ALFF, or fALFF, between violent video game group and the control part, indicating that long time exposure to violent video games won’t significantly influence spontaneous brain activity, especially the core brain regions such as execution control, moral judgment and short-term memory. This implies the adverse impact of violent video games is exaggerated.

## Introduction

With the rapid development of video game industry, video game plays an important part in our daily life. According to CNNIC (China Internet network information center), until June 2017, there are 751 million cyber citizens in China, one fifth of the world cyber citizens, and the amount of cyber citizens who play online video games is 421.64 million in China, and still keep increasing. It should be noted that most video games contain violent content (Yoon and Somers, 2003). Moreover, violent crimes associated with violent video game happened from time to time, which leads to a heated discussion among violent video games and aggression.

Many behavioral researches have proved that long time exposure to violent video games is associated with aggression. Violent video games contains lots of shooting, stabbing, boxing and kicking actions to hurt other game characters to avoid being killed or to achieve the goal (Anderson et al., 2010), which prompts the increasing of aggressive attitudes, beliefs and personality (Anderson and Dill, 2000) and leads to low level of helping behavior and prosocial behavior (Bushman and Anderson, 2009; Gentile et al., 2009; Anderson et al., 2010). Specifically, Engelhardt et al. (2011) using event-related potential technique and found that, compared to playing non-violent video game, the same duration of playing violent video game for 25 min leaded to desensitization to real life violence, and higher level of aggression in the competitive reaction time (CRT) task. Arriaga et al. (2014) measured the involuntary pupil dilation responses (PDR) after playing the game, and it turned out that participants in the violent video game conditions showed lower PDR to the victims of violence than participants in the non-violent game condition, which indicated violent video game players were not sensitive to violent scene. In addition, Anderson and Dill (2000) found that compared to non-violent video game, violent video game triggered players’s aggressive cognition, resulting in higher aggression level in the CRT task. Study launched by Bartholow and Anderson (2002) proved that only 10 min of violent video game play could result in more aggressive behavior than non-violent video game play. According to General Aggression Model (Anderson and Bushman, 2002), excessive violent video game use can cause long time effect, that is, repeatedly exposure to violent video game for months or years will reinforce individuals’ aggressive opponent in their believes and personality, transferring the state aggression to trait aggression. This indicates that long time exposure to violent video game may lead to permanent changes embodied in their brain changes of violent video gamers.

Neuroimaging studies have deepened our understanding of violent video game effects. There are many task-functional magnetic resonance imaging (fMRI) researches about violent video games and aggression. According to Gentile et al. (2016), there are three types task-fMRI researches. First, two or more groups of participants with different violent video game experiences are recruited to complete the cognitive or affective tasks, while their fMRI data is collected. Specifically, Mathews et al. (2005) found that high media violence participants displayed lower anterior cingulate activation during the stroop task than low media violence participants. Second, participants are required to play violent video games, and their brain activation in the different part of game play (e.g., violent vs. non-violent) are compared. Moreover, Weber et al. (2006) found an active suppression of emotional areas [rostral anterior cingulate cortex (ACC) and amygdala] as well as increased activity in planning and control areas (dorsal anterior cingulate cortex), specifically around the time of firing a weapon. Third, participants are randomly assigned to play the violent or non-violent video game for around 25 min, and then perform some cognition or affective tasks while fMRI data is recorded. Wang et al. (2009) examined brain activity during a Counting Stroop task and during an Emotional Stroop task and found that participants who had just played a violent game displayed relatively lower functional connectivity between the left dorsolateral prefrontal cortex and the ACC during the Counting Stroop task. Similarly, Hummer et al. (2010) found that playing a violent video game decreased activity in prefrontal cortex regions during a Go-No Go task.

In addition, quite a few fMRI researches about video games used to focus on the addiction aspect. Animal electrophysiological researches and human functional imaging researches have all proved that there existed a reward system, i.e., the dopamine neural circuit (O’Doherty, 2004; Bressan and Crippa, 2005; Knutson and Cooper, 2005; Schultz, 2006; Delgado, 2007; Hikosaka et al., 2008; Haber and Knutson, 2010). Plenty of researches have repeated this statement. For example, Ko et al. (2013) found that there was an overlap between brain activities of default mode network (DMN) and game addiction in game addiction group. Hoeft et al. (2008) found that males showed greater activation and functional connectivity compared to females in the mesocorticolimbic system, which may be attributable to higher motivational states in males, as well as gender differences in reward prediction, learning reward values and cognitive state during computer video games. It is suggested that long time exposure to violent video games could also reinforce this system, as rewarding is quite the inherent nature of game (Koepp et al., 1998; Mathiak et al., 2013).

In conclusion, abundant researches have showed that long time exposure to violent video games is highly associated with certain brain changes in many perspectives. Still, it should be noted that findings in behavioral researches can be confounded by subjects’ consciousness, task fMRI researches used to focus on one aspect of brain changes (i.e., brain changes related to the cognition or emotion), and concerning addiction researches are still quite few (Kim and Kim, 2010; Van Rooij et al., 2011; Zhu et al., 2015). Besides, spontaneous brain activity associated with violent video game exposure has not yet been well understood. Thus the resting-state fMRI study could be employed to investigate the long time effect of violent video games. To sum up, the hypothesis in this research is long time exposure to violent video games is associated with abnormal spontaneous brain activities.

Resting-state fMRI technique refines to measure the BOLD signal in the resting mood, the spontaneous brain activities without information import or export (i.e., performing any task). The spontaneous fluctuations that occur during the resting state reveal the intrinsic functional architecture of the brain and are related to extrinsic behavior performance (Fox and Raichle, 2007). What’s more, the task-free condition makes it more straightforward to investigate spontaneous brain activities that are related to behavioral performances (Bing et al., 2013). Thus, the resting state blood oxygen level-dependent signal has an edge in identifying the underlying neural basis of long time exposure to violent video games. The amplitude of spontaneous low-frequency fluctuation (ALFF) is widely used in indicating the extent of spontaneous neuronal activity (Zang et al., 2007), with high test–retest reliability (Zuo and Xing, 2014). Fractional ALFF is the ratio of power spectrum of low-frequency to that of entire frequency range, which is thought to be more robust with higher sensibility and higher specificity (Zou et al., 2008). The intention of selecting two indicator is to maintain the reliability of our results. In this way, it is applicable in identifying the potential neural circuit of long time effects of violent video games. Using these methods, the present study examined the spontaneous brain activity affected by violent video game exposure.

## Materials and Methods

### Participants

Fifty-two right-handed males (21.08 years, *SD* = 1.76, range: 17 ∼ 27 years) were recruited in this study, who played violent video games (i.e., games contain shooting, killing, slashing, and wrestling, like League of Legends, Counter-Strike, Grand Theft Auto, Warcraft, Cross Fire) more than 10 h a week in recent 3 months, while the non-violent video game group refers to participants who did not play violent video game at all. They were recruited through flyers posted across the campus of Southwest University, China. All of them had normal or corrected-to-normal vision and had no history of psychiatric or neurological disorders based on self-report. Before the experiments, all participants were informed of their right to privacy, and that they could quit the experiments anytime. After the experiments, each of them was paid $10 for their participation. Written informed consent was obtained from each participant, and this study was approved by the Administration Committee of Psychological Research at Southwest University. Three participants were removed from the sample due to excessive head motion during data preprocessing, and the remaining 49 participants were included in the formal data analysis.

#### Video Game Questionnaire

Video game questionnaire (Anderson and Dill, 2000) is used to select participants with different video game experience. Participants were asked to list three favorite video games, the number of hours they played each game in a week, and then rate the violence of their content and graphics (from 1 = not at all to 7 = extremely). High score indicates high video game experience. The questionnaire showed good reliability with internal consistency coefficient 0.91. This is to make sure the two groups, violent video game group and the control group are selected correctly.

#### Buss-Perry Aggression Questionaire (BPAQ)

This questionaire was used to measure trait aggression, which was compiled by Buss and Perry (1992). BPAQ consists of 29 items and four subscales: physical aggression; verbal aggression; anger and hostility. Participants rate themselves on each statement, on a scale of 1, extremely uncharacteristic of me, to 5, extremely characteristic of me. The higher the score, the higher the level of aggression. Among young adults, internal consistency alpha coefficients of BPAQ range from 0.55 to 0.94 in China. Additionally, the BPAQ has test–retest reliability coefficients around 0.81. Its construct validity is supported by other self-report methods of personality traits. In our study, the internal consistency alpha coefficients of BPAQ was 0.832. This questionaire is to investigate whether there exists significant difference in trait aggression between violent video game group and the control group.

### Image Acquisition

Participants were scanned in a 3.0 Tesla Siemens Trio scanner (Siemens Medical, Erlangen, Germany). First, high-resolution T1-weighted structural images were acquired sagittally. The scanning parameters were as follows: 1900/2.52 ms (repetition time/echo time), 1 mm (thickness), 176 slices; 256 mm × 256 mm (field of view), 900 ms (inversion time), 9° (flip angle) and 1 mm × 1 mm × 1 mm (voxel size). Then, functional images were obtained using a T2-weighted gradient recalled echo planar imaging sequence with the following parameters: 25 axial slices; slice thickness = 5 mm; repetition time = 1500 ms; echo time = 29 ms; image matrix = 64 × 64; field of view = 192 mm × 192 mm; flip angle = 90°; voxel size = 3 mm × 3 mm × 3 mm; volumes = 200. During the resting state scanning, participants were instructed to stay awake with their eyes closed and not think about anything in particular.

### Data Preprocessing

The preprocessing of resting state fMRI images was performed using a toolbox for Acquired Data were preprocessed using Data Processing Assistant for Resting-State fMRI (DPARSFA^1^) based on SPM8^2^, which was run on the matlab R 2009a software^3^ (MathWorks Inc.). The preprocessing steps were as follows: (1) Images from the first 10 volumes at the beginning of the resting state scanning were discarded to eliminate magnetic saturation effects; (2) the remaining 190 images were corrected for slice timing and head motion correction to adjust the time series of the images (head motion was <2.5 mm of translation along any axis and <2.5° of angular rotation along any axis). 27 violent video gamers and 22 non-violent video game participants were valid in the present study; (3) the structural images were coregistered to the mean functional image and were subsequently segmented as gray matter, white matter and cerebrospinal fluid employing the new segment method; (4) each functional image was normalized to the standard Montreal Neurological Institute (MNI) space with the application of DARTEL (diffeomorphic anatomical registration through exponentiated Lie algebra) (3 mm × 3 mm × 3 mm resampling); (5) after normalization, spatial smoothing was performed with an 8 mm full-width-half-maximum Gaussian kernel; (6) nuisance linear regression was performed with the white matter, cerebrospinal fluid and 6 rigid body head motion parameters; (7) the linear trends were removed, and finally, the images were temporally band-pass filtered (0.01–0.08 Hz) to reduce low-frequency drift and high-frequency noise (Biswal et al., 1995).

### Data Analysis

#### ALFF, fALFF Calculation

Amplitude of low-frequency fluctuations calculations were performed using the Resting-State fMRI Data Analysis Toolkit (version 1.8; Song et al., 2011). The ALFF is defined as the strength of regional spontaneous fluctuations of a given brain region. According to the methods proposed by Zang et al. (2007), time series in each voxel was transformed to a frequency domain with a fast Fourier transformation, and the power spectrum was obtained. Area under the peak point can be considered as the energy of signals. Then, the square root of the power spectrum was calculated and each voxel in 0.01–0.08 Hz was averaged. This averaged square root was considered to be the ALFF. For standardization purposes, the ALFF value of each voxel was divided by the global mean ALFF value to normalize the global volume effects (Zang et al., 2007). Because low-frequency fluctuations in the gray matter are higher than those in the white matter (Biswal et al., 1995; Wei et al., 2012), only ALFFs in the gray mask were calculated.

Fractional ALFF is the standardized value of ALFF, it is a ratio of the power of each frequency at the low-frequency range (0.01–0.08 Hz) to that of the entire frequency range (0–0.25 Hz). Same as ALFF, the *Z* scores of fALFF were calculated by using the fALFF value to minus the mean value of global mean value and then divided by the standard deviation (Zou et al., 2008).

### Statistical Analysis

First of all, to see whether our grouping is correct or not, we examined difference of violent video game experience between violent video game experience group and the control group by performing an independent sample *T*-test.

To compare the results of spontaneous brain activity during resting-state fMRI and trait aggression measured by BPAQ and better understand our results, we also conducted the independent samples *T*-test between the BPAQ score of violent video game group and that of control group.

To investigate the differences in spontaneous brain activation between groups, we performed two samples *T*-test on both ALFF and fALFF. AlphaSim correction was conducted for multiple comparison correction.

## Results

Independent samples *T*-test based on violent video game experience showed that there were significant difference (*t* = 4.00, *p* < 0.001) between violent video game group (41.40 ± 20.85) and the control group (18.53 ± 18.66).

Independent samples *T*-test based on BPAQ score showed that there were no significant difference (*t* = 0.73, *p* = 0.47) between violent video game group (68.89 ± 12.07) and the control group (66.42 ± 12.41), and there were no significant difference on physical aggression (*t* = 1.01, *p* = 0.31); anger (*t* = 0.07, *p* = 0.94) and hostility (*t* = -0.76, *p* = 0.47). However, there were significant difference on verbal aggression (*t* = 2.01, *p* = 0.045) between violent video game group (14.36 ± 3.48) and the control group (12.21 ± 3.97).

### Group Differences in ALFF Data

**FIGURE 1:**
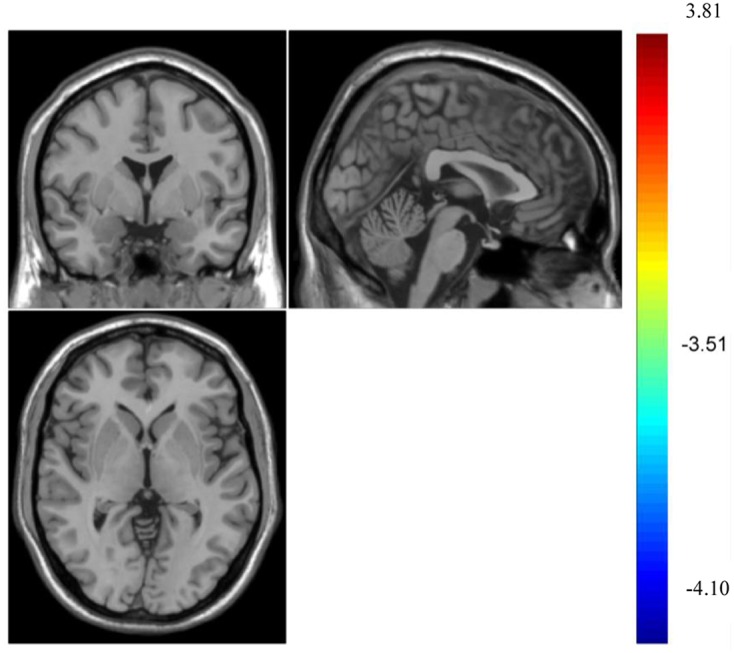
Result of amplitude of low-frequency fluctuation (ALFF) after Alphasim correction, with voxel size *p* < 0.001, cluster size = 13, *p* < 0.05. No clusters were found.

To examine the spontaneous brain activities between group of violent video games and group of non-violent video game, we performed two samples *T*-test. The results showed that there was no significant difference between violent video gamers and their counterparts in ALFF, AlphaSim correction was performed with voxel size *p* < 0.001, and cluster size *p* < 0.05, voxel = 13, see **Figure 1**. There was no significant difference between violent video game group and the control group in fALFF, either.

## Discussion

Resting-state fMRI were employed to investigate whether long time exposure to violent video games is related to abnormal spontaneous brain activities.

First, two samples *T*-test on violent video game experience showed that there were significant difference between violent video game group and the control group. The violent video game experience of former group is significantly higher than that of the control group, which proved that our grouping is correct and effective. This is the premise of further analysis.

For ALFF and fALFF, there were no significant difference between violent video game group and the control group, which is consistent with the result of two samples *T*-test on BPAQ total score. The two group are not remarkably different on spontaneous activities or trait aggression. This is against our former hypothesis.

As mentioned in former fMRI researches (Young et al., 2010; Molenberghs et al., 2015; Gentile et al., 2016), ACC, orbitofrontal cortex (OFC), temporo-parietal junction (TPJ) are the core regions related to aggression and moral judgement.

It should also be noted that ACC, OFC, and TPJ are the significant part in the DMN (the default mode network). DMN is distinctly important in emotion-processing, monitoring environment changes, self-introspection, maintaining self-awareness, and also extracting episodic memories (Immordino-Yang et al., 2012). Previous researchers have found that aggression is highly associated with misfunction in ACC (van Meel et al., 2007; Olvet and Hajcak, 2008). ACC can predict aggressive behavior, and people with injured ACC also behave in an aggressive way (Boes et al., 2008; Ducharme et al., 2011). As a crucial part in limbic system, ACC has rich functional connectivities with many regions, like prefrontal gyrus, hippocampal gyrus, parietal cortex, and hypothalamus. Specifically, ACC plays an important part in executive control (Awh and Gehring, 1999). Our findings suggested that individuals long-time exposed to violent video games did not show neuropsychological evidence of weak self-monitoring and self-control. OFC is one of the vital regions in moral sensitivity, which is quite important in judging the moral attributes of certain behaviors as morally justified or morally unjustified (Moll et al., 2007; Decety and Porges, 2011). OFC will be activated when individuals try hard to restrain the feeling of disgust and pain to adjust their feelings (Hooker and Knight, 2006). It is also a core node in moral sensitivity. Our results illustrate that there is no misfunction of moral sensitivity in this area after long time exposure to violent video games. TPJ, a brain area where the temporal and parietal lobes meet, at the posterior end of the Sylvian fissure (Abu-Akel and Shamay-Tsoory, 2011), was involved in the process of the sense of agency and higher level social cognition, such as empathy, moral reasoning and theory of mind (Saxe and Wexler, 2005; Decety and Lamm, 2007; Young and Dungan, 2011). When the activity in TPJ was restrained by transcranial magnetic stimulation (TMS) technique. Individuals tended to consider the intentional hurting behavior as morally justified (Young et al., 2010). This illustrated that TPJ is quite important in moral judgement. Specifically, right TPJ is in the lime light and received more and more attention recently. It has been proved that right TPJ plays a vital part in reasoning other individuals’ mental status during moral judgement, then estimate their judgment as morally appropriate or inappropriate. Taken together, no spontaneous brain activities in these areas suggested that long time exposure to violent video games neither influence the function in DMN, nor results in inappropriate moral sensitivity, impaired ability to feel others’ pain and violence desensitization and violent behaviors, aggressive personality, subsequently.

What’s more, our research findings is supported by increasing number of literatures claiming that the severe effect of violent video games is overstated (Ferguson et al., 2008, 2017a,b; Decety et al., 2009; Ferguson, 2010; Collins and Freeman, 2013; DeCamp and Ferguson, 2017; Gao et al., 2017; Hilgard et al., 2017; Szycik et al., 2017a,b). Ferguson et al. (2017b) allocated young adult players randomly to either play violent game, non-violent game, or to be given the choice between several violent and non-violent games, and then the ice water task was performed on each individuals to measure the aggression level, as well as stress levels and hostility. Results showed that there were no difference between different game conditions on hostility, stress, or aggression, indicating no evidence that violent video games can contribute to aggression. Gao et al. (2017) found that the perception of others’ pain were not significantly different in brain regions between VG (violent video gamers group) and NG (non-violent video gamers group), the desensitization effect of VVGs was overrated. Another research (Szycik et al., 2017b) also proved that there were no significant differences in brain responses when viewing pictures depicting emotional and neutral situations with and without social interaction, 15 excessive users of violent games and control subjects matched for age and education included. They suggested that the impact of violent video games on emotional processing may acute and short-lived. In another research conducted by Szycik et al. (2017a), 28 male adult subjects were screened with excessive long time use of violent video games. They were examined in two experiments using standardized emotional pictures of positive, negative and neutral valence. No group differences were found even at reduced statistical thresholds which speaks against desensitization of emotion sensitive brain regions as a result of excessive use of violent video games.

Meta-analyses investigating the potential correlates between violent video games and violent behaviors were positive but with small effect size ranging between 1 and 4% (Anderson and Bushman, 2001; Sherry, 2001; Anderson et al., 2010) and has been challenged the existence of publication bias (Ferguson, 2007). What’s more, a recent meta-analysis (Hilgard et al., 2017) re-examined another meta-analysis with the opinion of associations between violent video games and aggression (Anderson et al., 2010), in this present meta-analysis, a developed techniques was employed to detect the publication and analytic bias, as well as adjusting effect sizes. The results are quite different from the latter, with very little effect size. Our former research examining the difference in empathy for pain between violent video game group and non-violent video game group also showed evidence against the idea that long time exposure to violent video games will cause desensitization effect.

One can speculate from the above findings that there is no causal link between violent video games and aggression. This is mainly because, firstly the cause of aggression cannot be simply illuminated. Many environmental factors, such as childhood trauma, family background, contribute to aggression, mutually. For instance, research conducted by DeCamp and Ferguson (2017) found that the relationships between violent video games and aggressive behavior reduced to trivial effect size when controlled factors like parental attachment, youth disclosure, home yelling, home violence. Secondly, individuals are not the “blank slate” players. All the influence exerted by violent video games, or other outside forces is moderated by the characteristics of the player (e.g., cognitive styles, gender, different criteria of moral judgement). It has been proved that different motivations for choosing the same violent video game could resulting in different consequences, some of individuals may choose one violent video game as a way to release their stress, while others may choose the same violent games as a way to be able to conduct violent behavior and enjoy violence. As a result, the former one will probably be able to adjust himself to a better mood while the latter one is more likely to ruminate into violent content and causing serious consequences (Bowman and Tamborini, 2015; Ferguson et al., 2017a).

In China, the risk factors are mainly biological causes like gene defect, IQ, and gender; family causes like family structure, parent–child relationship, parental supervision; social factors, especially the function of peer pressure and bullying. These factors will influence juvenile delinquency, more in a combined way than solely. Violent video games is not the primary cause of aggression. According to General Aggressive Model (GAM), after gameplay, the oppression, provocation and clues about aggression will affect individuals’ mental process, impelling the development of aggressive cognition, emotion and behavior. Moreover, repeated exposure to violent video games could reinforce this loop and the aggressive personality is developed. However, this theory doesn’t fully explained the relationship between violent video games and aggression, players are seen as passive, negative receiver to what are put on them. It failed to pay enough attention to the subjective initiative of players. Our research emphasis the necessity to develop more sophisticated model, as well as advanced methodologies, to better explain the possible factors and pathways will lead to aggression.

There were also deficiencies in our study. First of all, our participants are all selected from college, whether our findings can apply to different groups remains uncertain. What’s more, given the fact that female violent video game players are quite few, our research target mainly focused on the males. The resting state brain activities in females and in male–female comparison worth further investigation. And further researches should also pay more attention to other potential aspects relating to aggression in more specific and sophisticated way.

## Conclusion

On the whole, our results suggested that there is no strong link between long time exposure to violent video games and spontaneous brain activity, didn’t show any neuropsychological evidence of aggression, enhanced our understanding to the relationship between long time exposure to violent video games and aggression.

## Ethics Statement

Ethical approval: All procedures performed in studies involving human participants were in accordance with the ethical standards of the institutional and/or national research committee and with the 1964 Helsinki declaration and its later amendments or comparable ethical standards.

Written informed consent was obtained after detailed explanation of the study protocol, which was approved by the Ethics Committee of Southwest University. The Institutional Review Board at Southwest University (SWU) in Chongqing, China approved this consent procedure. Written informed consent was obtained from all participants. The Institutional Review Board at SWU approved all procedures.

Informed consent: Informed consent was obtained from all individual participants included in the study.

## Author Contributions

Conceived and designed the experiments: XG and WP. Performed the experiments: WP, XG, SS, FL, and CL. Analyzed the data: WP, SS, FL, and CL. Wrote the paper: WP and XG.

## Conflict of Interest Statement

The authors declare that the research was conducted in the absence of any commercial or financial relationships that could be construed as a potential conflict of interest.

## Footnotes

1 http://restfmri.net/forum/DPARSF

2 http://www.fil.ion.ucl.ac.uk/spm/software/spm8/

3 http://www.mathworks.com
